# A New Species of *Euphlyctis* (Anura: Dicroglossidae) from Barisal, Bangladesh

**DOI:** 10.1371/journal.pone.0116666

**Published:** 2015-02-04

**Authors:** Mohammad Sajid Ali Howlader, Abhilash Nair, Sujith V. Gopalan, Juha Merilä

**Affiliations:** 1 Ecological Genetics Research Unit, Department of Biosciences, University of Helsinki, Helsinki, Finland; 2 Molecular Ecology Laboratory, Rajiv Gandhi Centre for Biotechnology, Thiruvananthapuram, Kerala, India; BiK-F Biodiversity and Climate Research Center, GERMANY

## Abstract

A new species of the genus *Euphlyctis* is described from the Barisal district of Bangladesh and compared with its morphologically similar and geographically proximate congeners. The new species is highly divergent in comparison to other congeneric species on basis of sequence divergence in mitochondrial DNA gene sequences (ranging from 5.5% to 17.8% divergence). *Euphlyctis kalasgramensis* sp. nov. can be readily diagnosed by having the following combination of characters: snout-vent length (SVL) 30.44 – 37.88 mm, absence of mid-dorsal line, nostril–snout length 3% of SVL, nostril much closer to snout tip than eye, nostril–snout length 48% of distance from front of eyes to nostril, relative length of fingers (shortest to longest: 1 = 2 < 4 < 3), tibia length 59% of SVL, foot length 55% of SVL.

## Introduction

The genus *Euphlyctis* consists of six species distributed in western Asia, southern Arabian Peninsula, India to Afghanistan, Nepal, Malaya, and Sri Lanka [1]. In Bangladesh *Euphlyctis cyanophlyctis* is reported as one of the most common species distributed over both plains and hills [2–4]. However, when we examined the specimens from different museums in Bangladesh (accession numbers listed in S1 Table) to verify the distribution of *E*. *cyanophlyctis* in Bangladesh, we discovered that morphological characters of all of the examined specimens did not correspond to the published information of specimens from the type locality [5–7]. Recently, Alam *et al*. [8] reported the possible occurrence of several cryptic species in the *E*. *cyanophlyctis* species complex on the basis of high genetic divergence of mitochondrial gene sequences from the *E*. *cyanophlyctis* described from the type locality in Southern India near to Sri Lanka [1, 9].

Herein, we describe a new species, earlier recognized as *E*. *cyanophlyctis* from Bangladesh, which show high degree of genetic divergence from the species described from southern India by Joshy et al. [7]. Molecular phylogeny and morphological comparisons are provided to characterize the new species from the other species within the genus. Complete morphological description of the new species, along with notes on its natural history are also given.

## Materials and Methods

### Ethics statement

This study was conducted with appropriate permissions (CCF letter no. 22.01.0000.101.23.2012.681 for collecting specimens, CF memo no. 22.01.0000.101.23.2012 for transport) and guidelines from the responsible authority, the Forest Department, Ministry of Forest and Environment, People’s Republic of Bangladesh. The protocol of our collection and research were approved by the committee of the Wildlife Section of the Forest Department, Bangladesh, and strictly complied with the ethical conditions as dictated by it and the law of Wildlife Preservation & Security Acts, 2012 (Chapter 10, section 48). Collected specimens are not threatened species and they are not listed in IUCN Red list or by CITES. All specimens were collected from a small village in Bangladesh (22°45'N, 90°18'E), which is not a protected area.

### Taxa and specimens

**Figure 1:**
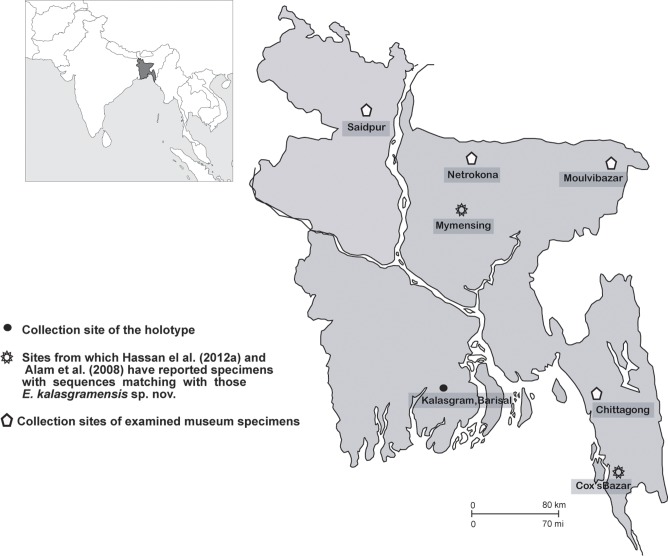
A map showing localities from where *Euphlyctis kalasgramensis* sp. nov. has been encountered.

The specimens (N = 15) were collected from Kalasgram, Barisal in Bangladesh (Fig. 1) The morphological measurements for all these specimens were taken and the specimens were deposited at the Finnish Museum of Natural History, Finland (MZH). Additional specimens used for morphological comparisons and their accession numbers are listed in S1 Table. Museum abbreviations include: MZH (Finnish Museum of Natural History, Finland), RGCB (Rajiv Gandhi Centre for Biotechnology, Kerala, India), MZD (Zoology Department, University of Chittagong, Bangladesh), and MHLB (Museum of Herpetology Laboratory Bangladesh, Ichamoti college, Dinajpur, Bangladesh).

### Morphological measurements

Measurements were taken with digital calipers to the nearest 0.02 mm. Characters measured follow the definitions of Islam *et al*. [10] and Kuramoto and Joshi [11] and include: SVL (snout-vent length), HL (head length), HW (head width), MN (distance from back of mandible to nostril), SL (snout length), MFE (distance from back of mandible to front of eye), MBE (distance from back of mandible to back of the eye), IN (internarial distance), IOD (interorbital distance), EN (distance from front of eyes to nostril), NS (nostril–snout length), EL (eye length), UEW (maximum width of upper eyelid), TD (tympanum diameter), TEL (tympanum–eye length), HAL (hand length), FAL (forearm length), THIGHL (thigh length), TL (tibia length), TFOL (length of tarsus and foot), FOL (foot length), and IMTL (inner metatarsal tubercle length). Description of webbing formula follows that of Glaw and Vences [12].

### Sequence analysis and phylogeny

For the genetic analysis, whole genomic DNA was extracted from muscle tissue (N = 15) using a silica-based method [13] and stored in -20°C. PCR amplification and sequencing of two mitochondrial DNA fragments (see below) was done using three pairs of primers (S2 Table). PCR reaction mix for both genes consisted of 5.72 μl of dH_2_O, 2 μl of 5x buffer, 0.08 μl of dNTP, 0.2 μl of Phire enzyme (Thermo Fisher) and 0.5 μl of each primer, in a total reaction volume of 10 μl. The PCR program comprised of a preliminary denaturation step at 98°C for 30s, followed by 34 cycles of 98°C for 10s, 55°C for 10s, 72°C for 30s and final extension at 72°C for 1 min. PCR products were purified by using ExoSap IT (USB Corporation, Cleveland, OH, USA) and sequenced at the Institute for Molecular Medicine Finland (FIMM). Sequence ambiguities were edited manually by aligning forward and reverse reads using the Geneious 5.6.5 program [14]. Obtained sequences were deposited in GenBank and the accession numbers are provided in S3 Table.

The available information of 16S and 12S rRNA genes sequences (N = 9) for all the known species in the genus *Euphlyctis* were obtained from GenBank. The 16S and 12S rRNA genes sequences were aligned using ClustalW as implemented in BIOEDIT [15, 16]. The final sequence length used for further phylogenetic analyses was 746bp. Pairwise genetic distances between the *Euphlyctis* were calculated using Mega v 5.5.6 [17] excluding the sites with indels. The phylogenetic analyses were performed using Maximum likelihood (ML) and Bayesian inference methods. The GTR + I + G substitution model was the most fitting nucleotide substitution model for the combined dataset and was used for ML analysis. For the ML analysis, branch support was evaluated by using 1000 bootstrap replicates [18] as implemented in Mega v 5.5.6 [17]. The Bayesian analysis was performed using MrBayes 3.1.2 [19] using the combined dataset of 16S and 12S gene fragments partitioned as separate gene fragments as they both are non-protein coding mitochondrial genes. The analysis was performed as a partitioned dataset with each gene fragment having the nucleotide substitution model (16S = SYM + G, 12S = GTR + G) chosen using the Akaike information criteria [20], implemented in jMODELTEST [21]. The Markov chain Monte Carlo runs were done for partitioned dataset for 1 million generations with sampling frequency of 100 and with each partition unlinked for the substitution parameters. Convergence of the runs was assessed by the average split frequency of standard deviations (<0.01) and by checking the potential scale reduction factors (~ 1.0) for all model parameters. The 25% of the trees were discarded as burn-in and the remaining trees were used to generate the 50% majority rule consensus tree and to estimate the Bayesian posterior probabilities.

### Nomenclatural acts

The electronic edition of this article conforms to the requirements of the amended International Code of Zoological Nomenclature, and hence the new names contained herein are available under that Code from the electronic edition of this article. This published work and the nomenclatural acts it contains have been registered in ZooBank, the online registration system for the ICZN. The ZooBank LSIDs (Life Science Identifiers) can be resolved and the associated information viewed through any standard web browser by appending the LSID to the prefix "http://zoobank.org/". The LSID for this publication is: urn:lsid:zoobank.org:pub:690B2A6B-EDF5-4ACA-BC77-485966954573. The electronic edition of this work was published in a journal with an ISSN, and has been archived and is available from the following digital repositories: PubMed Central, LOCKSS.

## Results

### Taxonomic treatment

Amphibia, Linnaeus, 1758

Anura Fischer von Waldheim, 1813

Dicroglossidae Anderson, 1871

Dicroglossinae Anderson, 1871

*Euphlyctis* Fitzinger, 1843

*Euphlyctis kalasgramensis* sp. nov. urn:lsid:zoobank.org:act:6720DE44-4BDD-470B-B3F7-0E15F1AD7763

**Etymology**. Species name is an adjective that refers to the Kalasgram, Barisal from where the holotype was collected.

**Holotype**. Adult male, MZH—3376, collected from Kalasgram (22°45'N, 90°18'E), Barisal, Bangladesh, collected by M. S. A. Howlader, May 22, 2012.

**Paratopotypes (n = 14)**. MZH—3377 (adult female), MZH—3390 (adult female), MZH—3378 (adult female), MZH—3379 (adult female), MZH—3380 (adult female), MZH—3381 (adult female), MZH—3382 (adult female), MZH—3383 (adult female), MZH—3384 (adult female), MZH—3385 (adult female), MZH—3386 (adult female), MZH—3387 (adult female), MZH—3388 (adult female), and MZH—3389 (adult female) collected from the same locality as the holotype by M. S. A. Howlader & P. Masud, May 23, 2012.

**Diagnosis**. The new species are characterized by a combination of the following characters: snout-vent length (SVL) of 37.88 mm in holotype male and 30.44–36.10 mm in Paratopotype females, absence of mid-dorsal line, nostril–snout length 3% of SVL, nostril much closer to snout tip than eye, nostril–snout length 48% of distance from front of eyes to nostril, relative length of fingers (shortest to longest: 1 = 2 < 4 < 3), tibia length 59% of SVL, foot length 55% of SVL.

**Figure 2:**
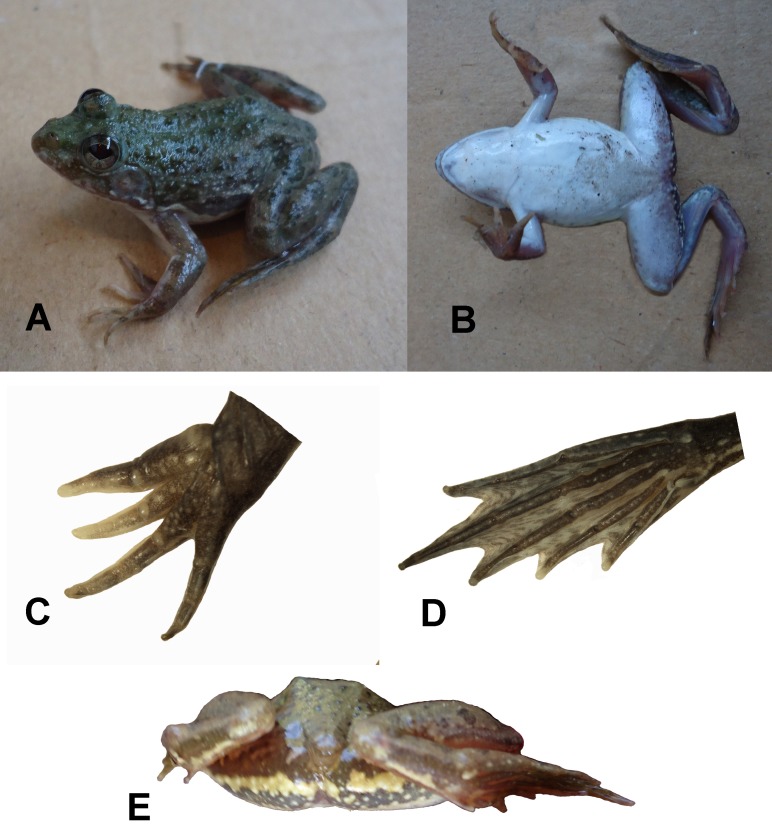
Photographs of *Euphlyctis kalasgramensis* sp. nov. (A) Dorso-lateral view of male (holotype) (B) Ventral view of male (holotype), (C) Ventral view of hand, (D) ventral view of foot, and (E) posterior view of thighs.

**Description of holotype (adult male; Fig. 2)**. Small sized frog (SVL 37.88 mm). Head large, triangular, broader than wide, HL 85% of HW, HW 38% of SVL, HL 32% of SVL, MFE 73% of HL, MBE 28% of HL. Snout nearly pointed in lateral view, SL 34% of HL, canthus rostralis indistinct, loreal region concave. Nostrils much closer to snout tip than to eyes, NS 47% of EN, NS 3% of SVL, EN 7% of SVL, nostrils rounded and very small, NS 53% of IN, MN 87% of HL. Eye large, EL 45% of HL, EL 14% of SVL, maximum width of upper eyelid greater than interorbital distance, IOD 64% of UEW, UEW 49% of EL, UEW 7% of SVL. Interorbital space convex, IOD 76% of IN. Tympanum round and tympanic fold distinct, TD 65% of EL.

Arms moderately long, robust, FAL 88% of HAL, FAL 24% of SVL, HAL 28% of SVL. Fingers small, free of webbing, tips rounded. Relative length of fingers, shortest to longest: 1 = 2 < 4 < 3, fingers lacking dermal ridge. Subarticular tubercles prominent, rounded, single tubercle per digit, supernumerary tubercles absent.

Hind limbs relatively long, TL 58% of SVL, THIGHL is equal to TL, FOL 54% of SVL, FOL 93% of TL, FOL 68% of TFOL. Toes long, thin, tips rounded, webbing between toes well developed [1(0.25), 2i(1), 2e(0.10), 3i(1.25), 3e(0.5), 4i(0.75), 4e(1), 5(0.75)]. Relative lengths of toes, shortest to longest: 1 < 2 < 5 < 3 < 4, a weak, indistinct fringe of skin on outer side of toe 5. Inner metatarsal tubercle elongated and compressed, present at base of toe 1, outer metatarsal tubercle is oval, minute, distinct, subarticular tubercles well developed, nearly oval. Dorsal surface rough with small tubercles and warts, warts present on upper side of the forelimb, thigh and tarsus, ventral surface smooth.

**Coloration in life**. Dorsal coloration of the body is grayish-brown with darker rounded spots (Fig. 2, S1 Video). Limbs with incomplete dark bands. There is a dark-edged white band on the hinder upper part of the thighs. Ventral surface is white.

**Coloration in preservative**. In preservative grayish-brown surface become either gray or grayish black. Color pattern of the body faded in comparison to live specimens.

**Measurements (in mm)**. Male (holotype): SVL 37.88, HL 12.20, HW 14.27, MN 10.66, SL 4.10, MFE 8.89, MBE 3.40, IN 2.31, IOD 1.75, EN 2.60, NS 1.22, EL 5.51, UEW 2.73, TD 3.60, HAL 10.56, FAL 9.28, LAL 7.75, THIGHL 23.13, TL 21.90, TFOL 29.83, FOL 20.40. Female (Paratopotypes, mean values): SVL 33.13, HW 12.24, HL 11.29, MN 10.21, MBE 3.44, IN 2.04, IO 1.7070, EN 22.09, EL 55.01, NS 1.02, SL 3.74, TD 3.32, UEW 2.62, HAL 9.69, FAL 6.78, LAL 55.53, TL 19.65, FOL 18.49, THIGHL 18.71, TFOL 27.17.

**Variation**. Adult females are larger than the males. In few females (two out of 14), ventral side pale yellowish with few tubercles like tiny dots in a regular arrangement.

**Distribution**. *Euphlyctis kalasgramensis* is known from the type locality as well as from all over the costal belt, hilly ranges, and central parts of Bangladesh. The species appears to have a wide distribution encompassing previously known distribution range of *E*. *cyanophlyctis* from Bangladesh (Fig. 1). It is possible that this species also occurs in adjacent areas in India.

**Natural history**. The new species has been found in ponds, rice and crop fields, and temporary pools. Specimens have been observed breeding in a shallow pond (22^th^ to 25^th^ May, 2012). Gravid females preferred choruses rather than individual or few calling males. Courtship was initiated when a gravid female approached the calling male and made a physical contact with it. The male stopped calling and suddenly jumped on the back of the female and clung to it by holding it below the armpits with the forearms and formed an axillary amplexus. The amplexed male also kicked other intruding males with its hind limbs. The amplexed pair moved to a small, shallow water pool over ground within the locality where the spawning occurred.

**Remarks and comparisons**. *Euphlyctis ghoshi* was described from Manipur province of India as *Rana ghoshi*, with a very distinct combination of characters including rounded head, head broad than long and snout length 15% of SVL [22, 23], which is not present in any other known species of the genus *Euphlyctis*. However, Chanda [23] suggested that *R*. *ghoshi* has close relation with *Euphlyctis*, *Lankanectes* and *Chrysopaa*. Still, mtDNA sequence information is not available to validate the generic status of *E*. *ghoshi*. Recently, Joshy *et al*. [7] reported two new species from the Western Ghats of India with molecular approaches and presented detailed morphological data for all the species in this genus. We have used those morphological characters to make qualitative comparisons between the new species described here and the other congeneric species (Table 1). To gain as good comparability as possible, we adopted trait definitions from Joshy *et al*. [7] and body proportions were used to probe the morphological divergence between species (cf. [24–27]). According to morphological ratios taken from Joshy *et al*. [7], the *E*. *kalasgramensis* sp. nov. is distinguished from other congeners occurring in southern Asia as follows: nostril–snout length 3% of snout-vent length (vs. nostril–snout length 6–8% of snout-vent length in *E*. *aloysii*, *E*. *mudigere*, *E*. *cyanophlyctis*, and *E*. *hexadactylus*), relative length of fingers, shortest to longest: 1 = 2 < 4 < 3 (vs. relative length of fingers: 2 < 4 < 1 < 3 in both *E*. *aloysii* and *E*. *hexadactylus*, 4 < 2 < 1 < 3 in both *E*. *mudigere* and *E*. *cyanophlyctis*), nostril–snout length 48% of distance from front of eyes to nostril (vs. nostril–snout length 88–95% of distance from front of eyes to nostril in *E*. *aloysii*, *E*. *mudigere*, *E*. *cyanophlyctis*, and *E*. *hexadactylus*), tibia length 59% of snout-vent length (vs. tibia length 48–50% of snout-vent length in *E*. *mudigere*, *E*. *cyanophlyctis*, and *E*. *hexadactylus*), foot length 55% of snout-vent length (vs. tibia length 47–50% of snout-vent length in *E*. *mudigere*, *E*. *cyanophlyctis*, and *E*. *hexadactylus*). Moreover, middorsal line is present in both *E*. *hexadactylus* and *E*. *aloysii* (vs. middorsal line is absent in *E*. *kalasgramensis* and other species of this genus; Table 1).

**Table 1:** Summary of qualitative diagnostic characters in *Euphlyctis kalasgramensis* sp. nov. and specimens of other congeners. Morphological ratios given as mean ± standard deviation over range.

	*E*. *kalasgramensis* sp.nov. (n = 15)		*E*. *cyanophlyctis* (n = 2)		*E*. *aloysii* (n = 24)		*E*. *mudigere* (n = 7)		*E*. *cyanophlyctis* (n = 19)		*E*. *hexadactylus* (n = 6*)*	
	X ± SD	Min-Max	X ± SD	Min-Max	X ± SD	Min-Max	X ± SD	Min-Max	X ± SD	Min-Max	X ± SD	Min-Max
NS:SVL	0.03 ± 0.005	0.02 – 0.03	0.06	0.06 – 0.07	0.06 ± 0.01	0.05 – 0.07	0.08 ± 0.02	0.05 – 0.10	0.07 ± 0.01	0.06 – 0.09	0.08 ± 0.01	0.06 – 0.10
EL:SVL	0.15 ± 0.01	0.14 – 0.17	0.12	0.12 – 0.13	0.10 ± 0.01	0.06 – 0.13	0.12 ± 0.02	0.08 – 0.14	0.13 ± 0.02	0.10 – 0.18	0.11 ± 0.01	0.09 – 0.12
AL:SVL	0.29 ± 0.01	0.28 – 0.30	---------	---------	0.24 ± 0.03	0.17 – 0.29	0.21 ± 0.05	0.15 – 0.28	0.26 ± 0.02	0.21 – 0.31	0.22 ± 0.03	0.16 – 0.25
TL:SVL	0.59 ± 0.01	0.58 – 0.61	0.47	0.47 – 0.48	0.48 ± 0.03	0.41 – 0.60	0.48 ± 0.03	0.45 – 0.52	0.50 ± 0.04	0.45 – 0.60	0.48 ± 0.01	0.46 – 0.49
FOL:SVL	0.55 ± 0.01	0.54 – 0.57	---------	---------	0.49 ± 0.03	0.39 – 0.55	0.47 ± 0.02	0.42 – 0.50	0.48 ± 0.05	0.41 – 0.61	0.50 ± 0.02	0.47 – 0.52
NS: EN	0.48 ± 0.01	0.47 – 0.49	0.91	0.86 – 0.96	0.92 ± 0.17	0.63 – 1.27	1.12 ± 0.54	0.43 – 2.00	0.95 ± 0.19	0.64 – 1.42	0.88 ± 0.15	0.67 – 1.06
Middorsal line	Absent		Absent		Present		Absent		Absent		Present	
Relative finger length	1 = 2 <4 < 3		4<2<1<3		2<4<1<3		4<2<1<3		4<2<1<3		2<4<1<3	
References	Present study		Present study		Joshy *et al*. [7]		Joshy *et al*. [7]		Joshy *et al*. [7]		Joshy *et al*. [7]	

**Figure 3:**
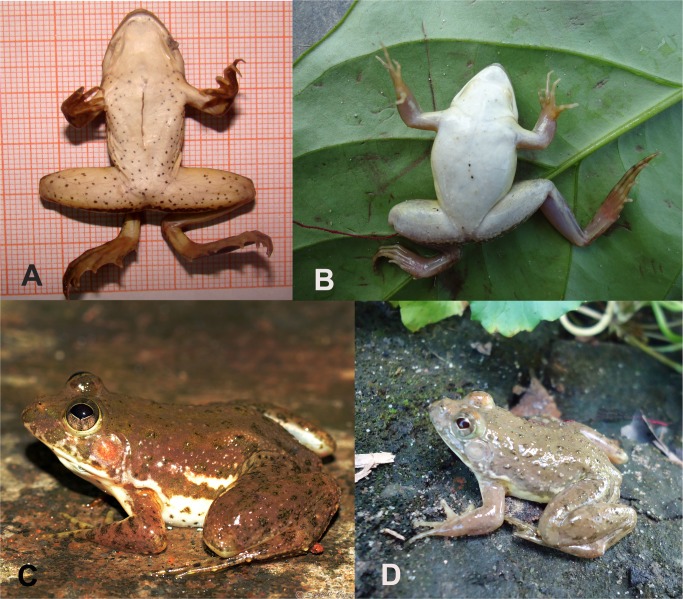
Photographs illustrating diagnostic characters of *Euphlyctis* species. Ventral views of (A) *Euphlyctis cyanophlyctis* (adult male, institutional collection’s specimen number: RGCB-5695) and (B) *Euphlyctis kalasgramensis* sp. nov. (adult male, institutional collection’s specimen number: MHL-B1001) (C) dorso-lateral view of *Euphlyctis cyanophlyctis* (adult male, institutional collection’s specimen number: RGCB-5695) and (D) dorso-lateral view of *Euphlyctis kalasgramensis* sp. nov. (adult male, institutional collection’s specimen number: MHL-B1001).

Moreover, we measured two *E*. *cyanophlyctis* from the type locality (Kerala, India), to compare them with the new species. The *E*. *cyanophlyctis* specimens that we measured (n = 2) were morphologically similar to *E*. *cyanophlyctis* reported by Joshy *et al*. [7] presented in Table 2. The specimens also show resemblance to the original description of *E*. *cyanophlyctis* bearing spotted ventral part which was described as one of the most notable character to distinguish this species from others [28]. *Euphlyctis kalasgramensis* differs from the specimens of *E*. *cyanophlyctis* from the type locality by having a whitish ventral coloration (*vs*. spotted in *E*. *cyanophlyctis*; Fig. 3), small snout-vent length: 31.13–36.10 mm in adult females (vs. SVL > 50 mm in *E*. *cyanophlyctis*). Also multivariate shape analyses provide evidence to suggest that *E*. *kalasgramensis* and *E*. *cyanophlyctis* from the type locality differ both in size and shape (S1 File).

**Table 2:** The mean genetic distances between *Euphlyctis* species clades (a–f) calculated using the Maximum composite Likelihood method, using a combined dataset of two mitochondrial genes (16S and 12S).

	Clade	(a)	(b)	(c)	(d)	(e)	(f)
(a)	Euphlyctis kalasgramensis						
(b)	Euphlyctis aloysii	0.178					
(c)	Euphlyctis mudigere	0.055	0.173				
(d)	Euphlyctis cyanophlyctis	0.057	0.182	0.046			
(e)	Euphlyctis hexadactylus	0.164	0.119	0.163	0.165		
(f)	Euphlyctis ehrenbergii	0.083	0.162	0.061	0.061	0.170	

**Figure 4:**
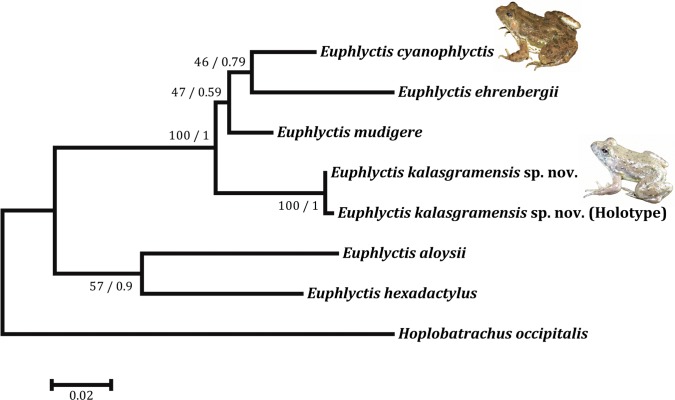
Phylogenetic relationships among available species in the genus *Euphlycti*s. Analysis is based on 746 bp mtDNA (16S and 12S gene sequences) showing the position of *Euphlyctis kalasgramensis s*p. nov. *Hoplobatrachus occipitalis* was used as an outgroup. Numbers on branches represent bootstrap support for Maximum-likelihood, and Bayesian posterior probability.

**Molecular phylogeny and genetic divergence from other species**. The combined dataset for the 16S and 12S gene fragments comprised of 746bp (474bp of 16S and 272bp of the 12S gene fragments). The Maximum likelihood and Bayesian methods resulted in similar phylogenetic trees (Fig. 4), *E*. *kalasgramensis* sp. nov. grouping with the both *E*. *mudigere* and *E*. *cyanophlyctis* and show high degree of genetic divergence from both the species (Table 2). *E*. *kalasgramensis* clade was strongly supported, in both Maximum likelihood (bootstrap value 100) and Bayesian analysis (posterior probability ~ 1, Fig. 4). The interspecific divergence of *E*. *kalasgramensis* from other species in the genus was high, ranging from 5.5% to 17.8% for the mitochondrial DNA sequences combined data set of 16S rRNA and 12S rRNA genes (Table 2). In contrast, the degree of intraspecific divergence was low (0.02% and 0.04% for 16S rRNA and 12S rRNA respectively).

## Discussion

*Euphlyctis cyanophlyctis* is one of the most common species in the *Euphlyctis*-genus and it exhibits high degree of morphological similarity with other species of the genus [7]. This species was first described by Schneider [28] as “*Rana cyanophlyetis*” and the type locality was mentioned as “India orientali” but later the type locality was considered to be “Tranquebar of southern India” [1, 9]. There are several species described within *E*. *cyanophlyctis* species complex, which are now grouped as synonymous species [1]. For example, *Rana bengalensis* Gray, 1830 is considered synonymous to *E*. *cyanophlyctis*. There is no detailed description of holotype available, the location of holotype is presently unknown and presumed to be lost [1]. However the figure of *R*. *bengalensis* from type locality "Bengal" [29] has more resemblance with *E*. *cyanophlyctis* from south India than with the *E*. *kalasgramensis* in its overall morphology, as well as in having a spotted dorsum [29]. *R*. *bengalensis* is also considered synonymous with *R*. *leschenaultii* described from type locality “Pondichéry and Bengale” [30], by Cantor [31]. The ventral parts of *Rana leschenaultia* are similar to the *E*. *cyanophlyctis* and this differentiates it from *E*. *kalasgramensis*. *Dicroglossus adolfi* which is presently considered as synonymous to *E*. *cyanophlyctis* was described by Günther [32] from type locality "Kulu and Simla, Himalaya (2400–4200 feet above the level of the sea)", India. This species was described initially mentioning similarity with *Occidozyga* and *Bombina* rather than *Euphlyctis*, but was later categorized as being synonymous to *E*. *cyanophlyctis* [33] However, the ventral portion in *Dicroglossus adolfi* is described having dark specks showing more similarity to *E*. *cyanophlyctis* than to *E*. *kalasgramensis* (whitish ventral coloration). According to Khan [34], *E*. *cyanophlyctis microspinulata* has first finger longer than second and presence of microscopical spinules scattered on body and limbs which clearly differentiates it from *E*. *kalasgramensis* (first and second fingers are equal and microscopical spinules are absent in *E*. *kalasgramensis*). The two color varieties of Sri Lankan *E*. *cyanophlyctis* were described by De Silva {[35]: “*Rana cyanophlictis variety fulvus*” with yellowish body and “*Rana cyanophlictis* variety *flavens”* with greenish body”} with no designation of types. The description of these specimens does not match with *E*. *kalasgramensis* as they differ in body coloration. *Rana cyanophlyctis* var. *seistanica* Nikolskii, 1899 was described from Iran with close similarity to Arabian *E*. *ehrenbergii* than to south Asian *E*. *cyanophlyctis*. *E*. *ehrenbergii* (Peters, 1863) had also been synonymized with *E*. *cyanophlyctis* for long time and was raised to current species status by Dubois [36]. This species has relatively larger body and dorsum with uniform green coloration [34], having more resemblance to *E*. *hexadactylus* than to *E*. *cyanophlyctis* or *E*. *kalasgramensis*.

Based on genetic comparisons, Kurabayashi *et al*. [37] found a new candidate species from the Western Ghats, which was formally recognized as *E*. *cyanophlyctis*. After this, Alam *et al*. [8] reported another candidate species from Bangladesh by making genetic comparison with all the known species in the genus. The results from these studies helped the identification and description of *E*. *mudigere* and *E*. *aloysii* which were in need of formal systematic description [7]. The genetic identities of *E*. *kalasgramensis* matches with the genetic information of the few specimens published in Alam *et al*. [8] that were left unnamed (haplotype abbreviation—Ecya-Ba1 and Ecya-Ba2) due to lack of sufficient number of specimens. We have provided here a detailed description of the morphology of the new candidate species identified by Alam *et al*. [8], which we describe here as *E*. *kalasgramensis* with a designated holotype submitted to the Finnish Natural History Museum and provided morphological differences between *E*. *kalasgramensis* and the congeners. The identification of cryptic taxa hidden under a common species name is becoming more and more frequent in this region [27, 38, 39] and future studies in this genus as well as in other related genera will help in identifying the species diversity of this poorly studied region.

## Supporting Information

S1 ARRIVE Checklist. (DOC)

S1 File Multivariate analyses of size and shape in *E*. *kalasgramensis* and *E*. *cyanophlyctis*. (PDF)

S1 Table Additional specimens examined. (DOC)

S2 Table Primers used for PCR amplification in the present study. (DOC)

S3 Table GenBank accession numbers of the sequences and collection localities of specimens used in this study. (DOC)

S1 Video Live specimen of *Euphlyctis kalasgramensis* sp. nov. (AVI)
