# Supplementary material for: A New Species of Euphlyctis (Anura: Dicroglossidae) from Barisal, Bangladesh
Source: PLoS One. 2015 Feb 4;10(2):e0116666. doi: 10.1371/journal.pone.0116666 (PMC4317184; doi:10.1371/journal.pone.0116666)

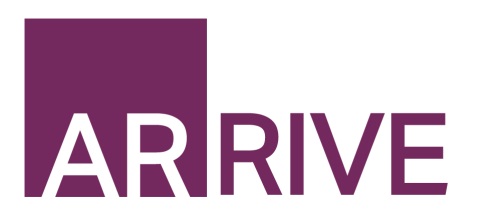


The ARRIVE Guidelines Checklist

Animal Research: Reporting In Vivo Experiments

Carol Kilkenny^1^, William J Browne^2^, Innes C Cuthill^3^, Michael Emerson^4^ and Douglas G Altman^5^

*^1^The National Centre for the Replacement, Refinement and Reduction of Animals in Research, London, UK, ^2^School of Veterinary Science, University of Bristol, Bristol, UK, ^3^School of Biological Sciences, University of Bristol, Bristol, UK, ^4^National Heart and Lung Institute, Imperial College London, UK, ^5^Centre for Statistics in Medicine, University of Oxford, Oxford, UK.*

|  | | ITEM | RECOMMENDATION | Section/ Paragraph |
| --- | --- | --- | --- | --- |
|  | 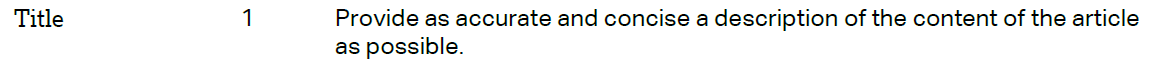 | | | Provided |
|  | 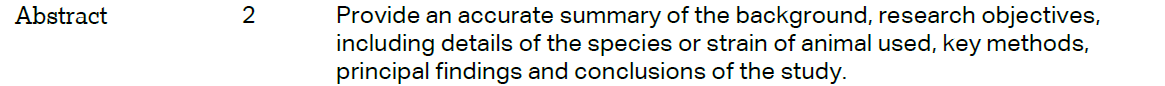 | | | Provided |
|  | INTRODUCTION | | |  |
|  | 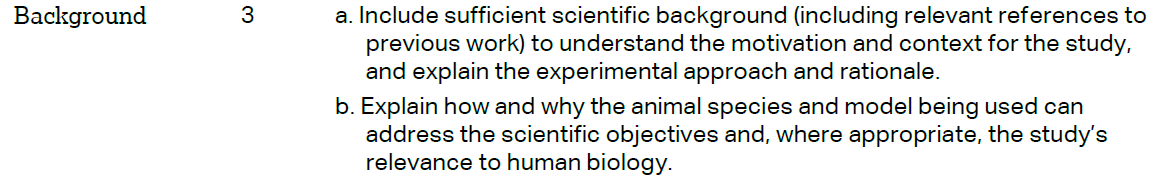 | | | Provided |
|  | 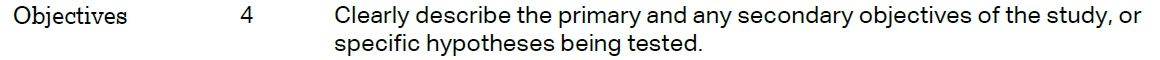 | | | Provided |
|  | METHODS | | |  |
|  | 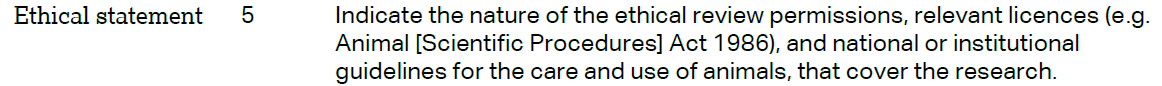 | | | Provided |
|  | 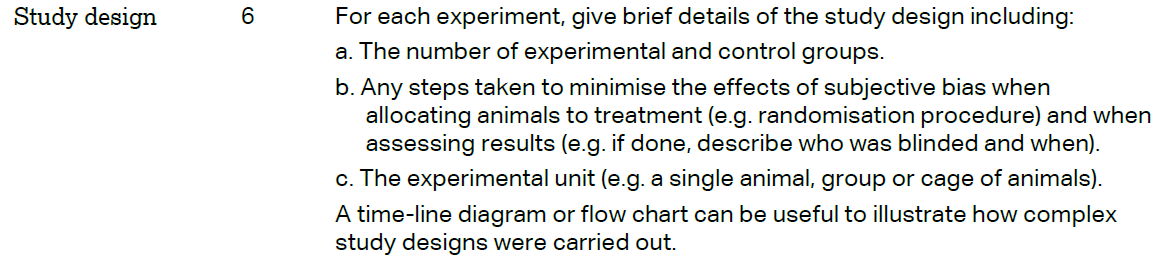 | | | Provided |
|  | 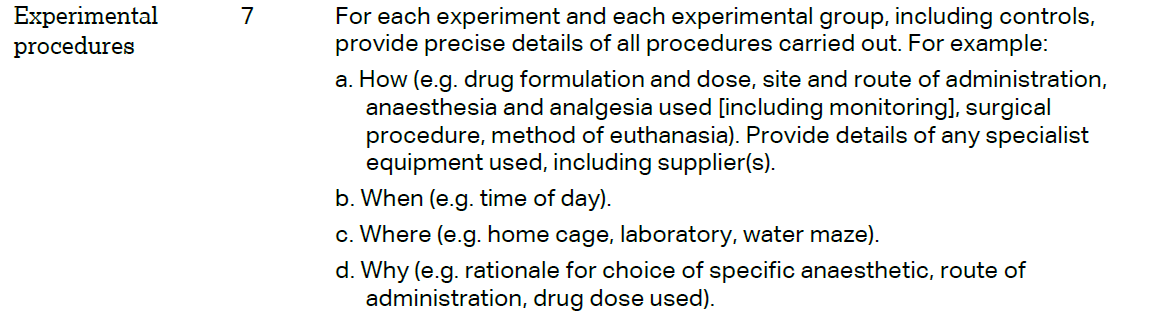 | | | Provided |
|  | 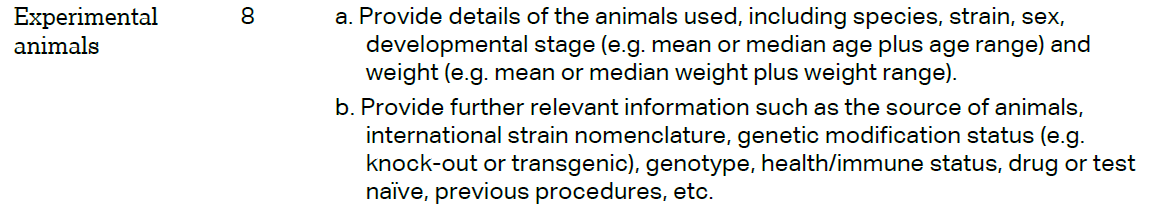 | | | Provided |

The ARRIVE guidelines. Originally published in *PLoS Biology*, June 2010^1^

|  | 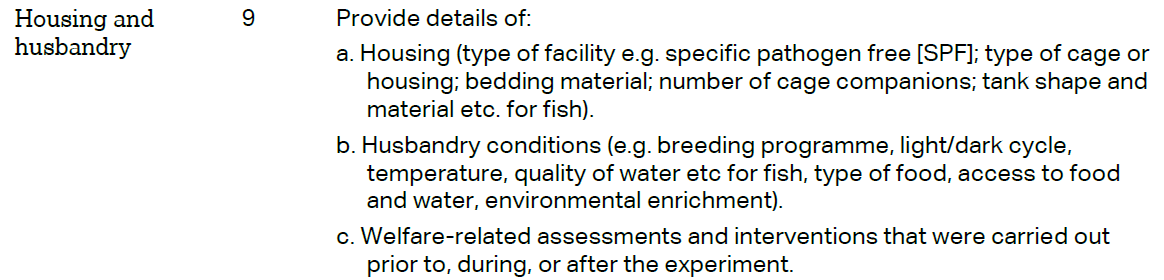 | Not applicable | |
| --- | --- | --- | --- |
|  | 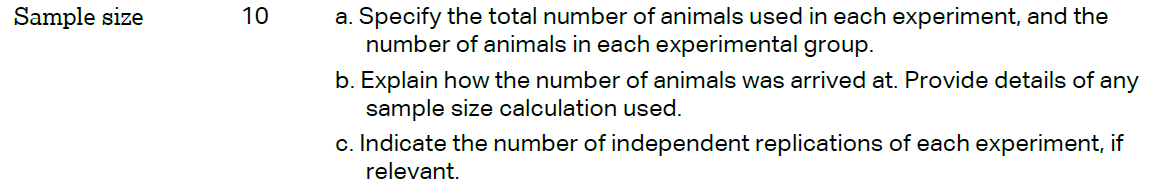 | Provided | |
|  | 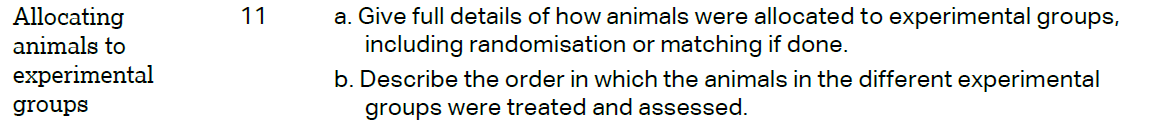 | Not applicable | |
|  | 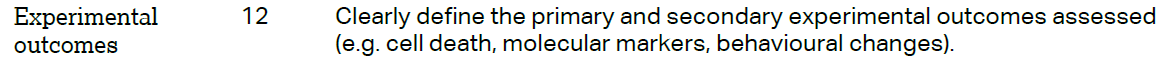 | Provided | |
|  | 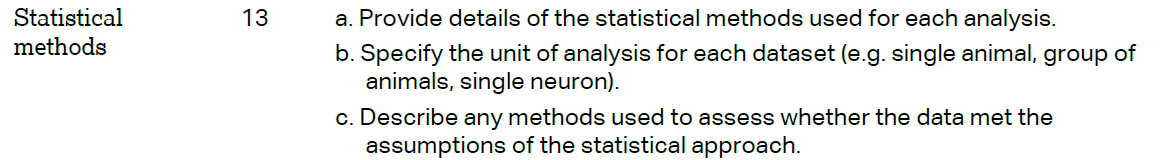 | Provided | |
|  | RESULTS |  | |
|  | 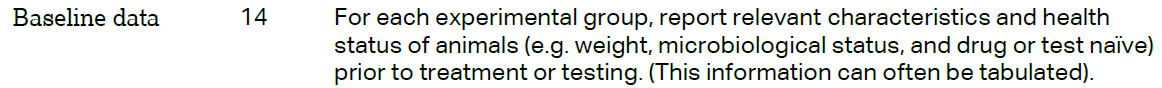 | Not applicable | |
|  | 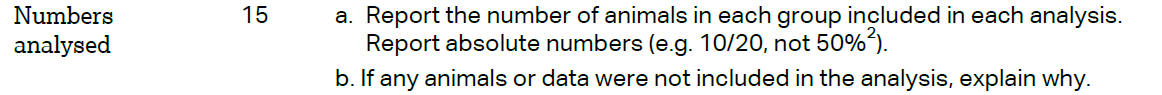 | Provided | |
|  | 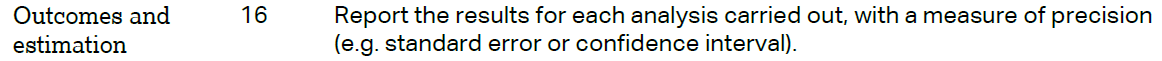 | Provided | |
|  | 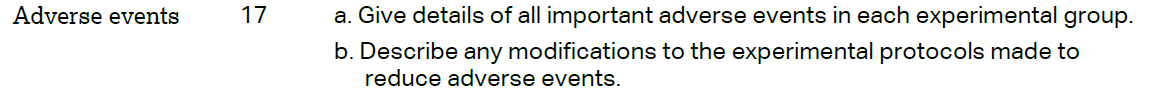 | Not applicable | |
|  | DISCUSSION |  | |
|  | 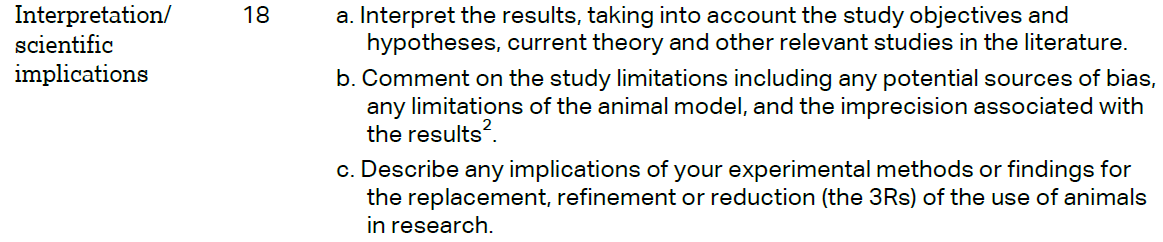 | Provided | |
|  | 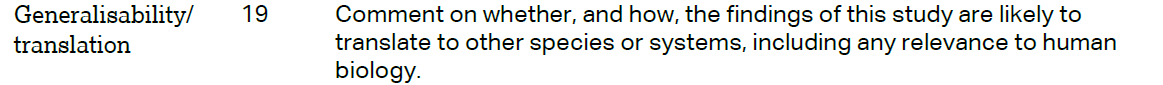 | Not applicable | |
| 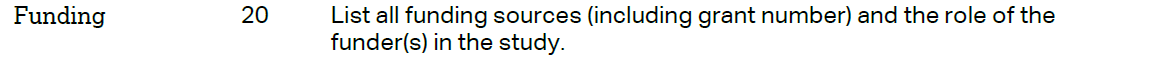 | | Provided |  |


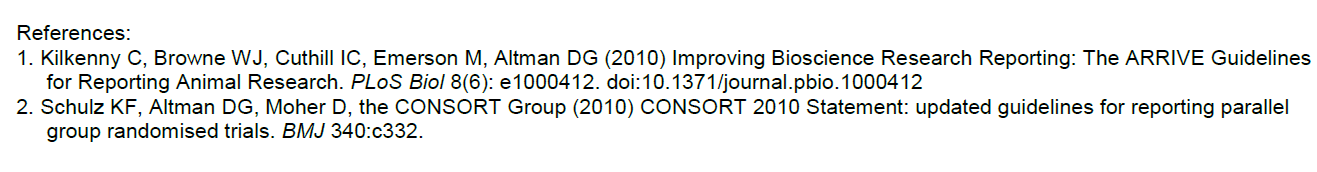

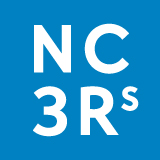

Supplement: S1 ARRIVE Checklist. — (DOC) [file pone.0116666.s001.doc]
