# Supplementary material for: A New Species of Euphlyctis (Anura: Dicroglossidae) from Barisal, Bangladesh
Source: PLoS One. 2015 Feb 4;10(2):e0116666. doi: 10.1371/journal.pone.0116666 (PMC4317184; doi:10.1371/journal.pone.0116666)
Supplement: S1 File — (PDF) [file pone.0116666.s002.pdf]

File S1. Multivariate analyses of size and shape in *E. kalasgramensis* and *E. cyanophlyctis*.

Although the *E. kalasgramensis* can be easily separated from *E. cyanophlyctis* on the basis of qualitative characters, we also conducted multivariate size and shape analyses based on available data (*E. kalasgramensis* n = 15*, E. cyanophlyctis* n = 2). A principal component analysis of 19 traits (see Material and Methods for trait list and definitions) revealed two significant (Eigenvalues > 1) Eigenvectors which explained 13.1% and 2.9% of variation in the data. The first axis was interpreted to be mainly a size axes as most of the traits loaded strongly and positively (with the exception of trait IN which loaded weakly negatively) on it- The second axis was a "shape" axis with strong (loadings: 0.45 – 0.93) positive loading on traits IN, TL and UEW, and strong (loadings: -0.60 – - 0.51) negative loadings on traits NS and EN. These two axes clearly separated the individuals of the two species as distinct clusters (Fig. SIF1). A Wilcoxon rank-sums test show that the mean values of both PC1 and PC2 scores differ significantly between the two species (PC1: ChiSquare = 4.56, d.f. = 1, P = 0.037; PC1: ChiSquare = 4.56, d.f. = 1, P = 0.037). A linear discriminant analyses of the data using forward selection procedure resulted in inclusion of five variables (EN, NS, LAL and TFOL) into the function which classifies all specimens correctly to their species group. Hence, these quantitative analyses – while based on limited data – support the view that apart from the qualitative criteria described in the main text, the two species can be differentiated from each others also solely on the basis of morphometric measurements. However, more individuals from multiple populations would be needed to verify this conclusion.

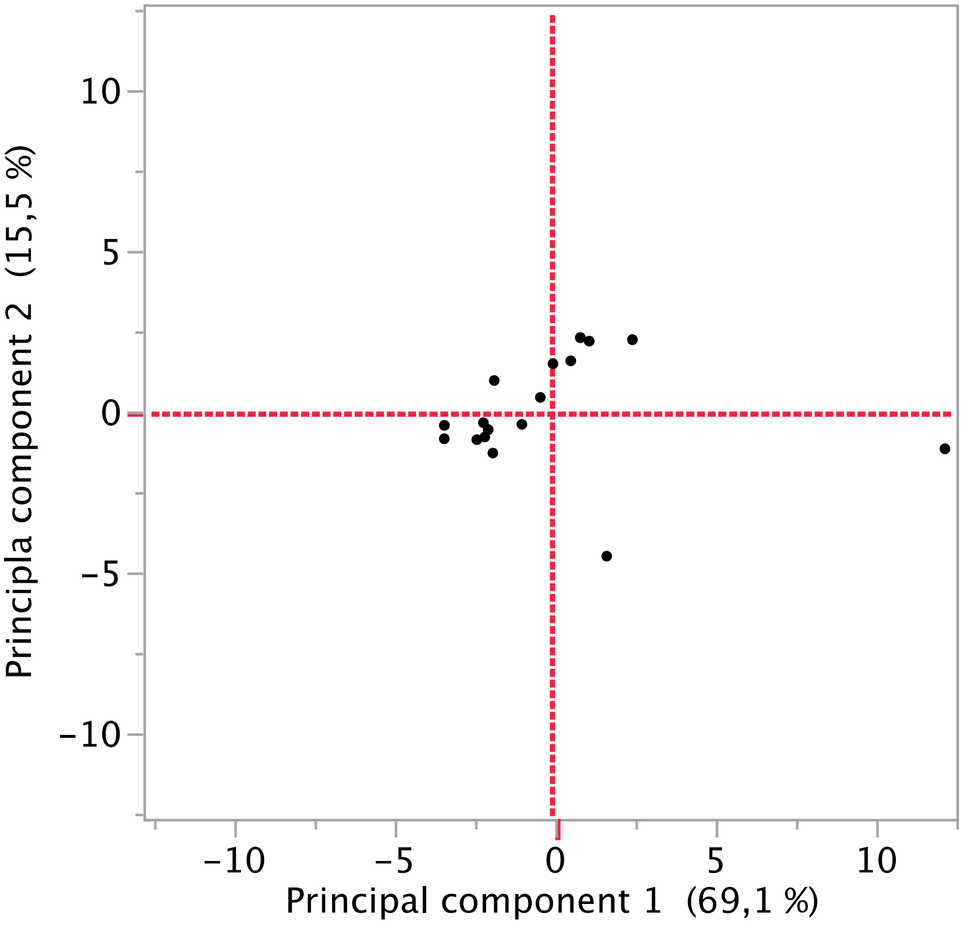

Figure S1. Principal component ordination of the *Euphlyctis* morphometric data. *E. kalasgramensis* individuals cluster close to the origo of the plot, and the two *E. cyanophlyctis* specimens from the type locality are the ones located in the lower right quarter of the figure.
