# Supplementary material for: A New Species of Euphlyctis (Anura: Dicroglossidae) from Barisal, Bangladesh
Source: PLoS One. 2015 Feb 4;10(2):e0116666. doi: 10.1371/journal.pone.0116666 (PMC4317184; doi:10.1371/journal.pone.0116666)
Supplement: S1 Table — (DOC) [file pone.0116666.s003.doc]

**Table S1.** Additional specimens examined.

***Euphlyctis kalasgramensis* (26 specimens):** Zoology Department, University of Chittagong: MZD 401–426. **Collection localities:** University of Chittagong campus, Hathazari, Bangladesh.

***Euphlyctis kalasgramensis* (11 specimens):** Museum of Herpetology Laboratory’ Bangladesh: MHLB1001–1011 **Collection localities:** Saidpur, Netrokona, Moulvibazar, Bangladesh.

***Euphlyctis cyanophlyctis* (2 specimens):** Rajiv Gandhi Centre for Biotechnology: RGCB-5695, RGCB -5696. **Collection localities:** Wayanad**,** Kerala, India.
