# Supplementary material for: A New Species of Euphlyctis (Anura: Dicroglossidae) from Barisal, Bangladesh
Source: PLoS One. 2015 Feb 4;10(2):e0116666. doi: 10.1371/journal.pone.0116666 (PMC4317184; doi:10.1371/journal.pone.0116666)
Supplement: S2 Table — (DOC) [file pone.0116666.s004.doc]

**Table S2. Primers used for PCR amplification in the present study.**

| **Gene** | **Primer** | **Sequence 5'- 3'** | **Reference** |
| --- | --- | --- | --- |
| **12S rRNA** | FS01 | 5'-AACGCTAAGATGAACCCTAAAAAGTTCT-3' | Sumida *et al.* [1] |
|  | R16M1 | 5'-GGGTATCTAATCCCAGTTTG-3' | Sumida *et al.* [1] |
| **16S**  **rRNA** | F51 | 5'-CCCGCCTGTTTACCAAAAACAT-3' | Sumida *et al.* [1] |
|  | R51 | 5'-GGTCTGAACTCAGATCACGTA-3' | Sumida *et al.* [1] |
|  | F | 5'- CGCCTGTTTATCAAAAACAT -3' | Palumbi *et al.* [2] |
|  | R | 5'- CCGGTCTGAACTCAGATCACGT -3' | Palumbi *et al.* [2] |

**References**

1. Sumida M, Kondo Y, Kanamori Y, Nishioka M (2002) Inter- and intraspecific evolutionary relationships of the rice frog *Rana limnocharis* and the allied species *R. cancrivora* inferred from crossing experiments and mitochondrial DNA sequences of the 12S and 16S rRNA genes. Molecular Phylogenetics and Evolution 25: 293–305.
2. Palumbi SR, Martin A, Romano S, McMillan WO, Stice L, et al. (1991) The Simple Fool’s Guide to PCR, Version 20, privately published document compiled by S Palumbi Dept Zoology, Univ Hawaii, Honolulu, HI.
