# Supplementary material for: A New Species of Euphlyctis (Anura: Dicroglossidae) from Barisal, Bangladesh
Source: PLoS One. 2015 Feb 4;10(2):e0116666. doi: 10.1371/journal.pone.0116666 (PMC4317184; doi:10.1371/journal.pone.0116666)
Supplement: S3 Table — (DOC) [file pone.0116666.s005.doc]

**Table S3.** GenBank accession numbers of the sequences and collection localities of specimens used in the present study.

| **Species** | **Collection site** | **Specimen accession number** | **GenBank accession number** | |
| --- | --- | --- | --- | --- |
|  |  |  | **16S rRNA** | **12S rRNA** |
| ***Euphlyctis aloysii*** | Bajpe, Mangalore,  Karnataka, India | BNHS 5123 | AB272606 | AB273171 |
| ***Euphlyctis mudigere*** | Mudigere, Western Ghats, India | BNHS 5127 | AB377109 | AB377110 |
| ***Euphlyctis cyanophlyctis*** | Mudikari, Karnataka, India | RBRL 03060702 | AB167938 | AB167910 |
| ***Euphlyctis hexadactylus*** | Mangalore,  Karnataka, India | RBRL 03060601 | AB167941 | AB167913 |
| ***Euphlyctis ehrenbergii*** | Yemen | MNHN 2000.649 | AY014367 | -------------- |
| ***Euphlyctis kalasgramensis* (Haplotype 1)** | Kalasgram, Barisal, Bangladesh | MZH 3376, MZH 3378, MZH 3379, MZH 3380, MZH 3381, MZH 3382, MZH 3385, MZH 3389, MZH 3390 | KP091862, KP091855, KP091866, KP091872, KP091868, KP091860, KP091857, KP091854, KP091863 | KP091878, KP091875, KP091872, KP091869, KP091879, KP091882, KP091876, KP091877 |
|  | Mymensingh,  Bangladesh {Ecya-Ba1; Alam et al.[8]} | ------------------- | AB272601 | -------------- |
| ***Euphlyctis kalasgramensis* (Haplotype 2)** | Kalasgram, Barisal, India | MZH 3377, MZH 3383, MZH 3384, MZH 3386, MZH 3387, MZH 3388 | KP091865, KP091856, KP091867, KP091858, KP091859, KP091864 | KP091873, KP091871, KP091870, KP091881, KP091880, KP091874 |
|  | Maymanshing,  Bangladesh  {Ecya-Ba2; Alam et al.[8]} | --------------------- | AB272602 | -------------- |
| *Note.* Museum abbreviations: RBRL, Rondano Biodiversity Research Laboratory of St. Aloysius College, India; BNHS, Bombay Natural History Society, India; MNHN, Muséum National d’Histoire Naturelle, Paris; MZH, Finnish Museum of Natural History, Finland. | | | | |
